# The Sterilisation of Undesirable Citizens

**Published:** 1922-11

**Authors:** G. T. K. Maurice

**Affiliations:** C.M.G.


### THE STERILISATION OF UNDESIRABLE CITIZENS.

By COLONEL G. T. K. MAURICE, C.M.G.

TURNING to the jury, Mr. Justice Roche said: "In my judgment, the medical profession of this country would be performing a public service if they studied earnestly the question of the feasibility of sterilising both men and women with tendencies such as the man before me has. To allow them to produce is breeding from the worst of all stock, and propagating disease and crime. I am expressing no opinion whether it is feasible or whether Parliament should pass such a measure. That depends on the examination of skilled persons as to the feasibility and risks attending."

Such a statement from a man in the position of Mr. Justice Roche is notable. It marks the rapid advance of modern thought on the question of birth-control. In the nineteenth century such ideas had hardly been formulated in the secrecy of the chamber; to-day they are uttered, with full sense of responsibility, from the bench. There is not need for the medical profession earnestly to study the feasibility of sterilising either men or women. It can be done by any man of ordinary surgical skill; and with but little danger to life, if the mere physical existence of the undesirable is to continue to be considered of importance. Not only can sterilisation be attained, but it can be attained without modification of sex characteristics. It is not necessary to make eunuchs of men nor sexless females of women to gain the end that persons likely to beget or to bear bad stock shall be deprived of the possibility of reproducing their kind. All that is wanting is that public opinion should be so enlightened—the last word is purposely used—as to agree that in the overcrowded state of the world it is not desirable that those persons who are not suited to communal life and are a burden on their community or are a danger to their community shall not be allowed to propagate; because, since black cats get black kittens, it is likely that a great part, even the most part, of their progeny will be a burden and no advantage to their community.

Up till now sentimentality and religion have joined forces to encourage the breeding not only of persons who cannot produce a sufficient portion of the national wealth, or give sufficiently efficient service to the community, to enable them to provide food and shelter for themselves and the families they produce, but even of persons who are a positive danger to the industrious and efficient by reason of their predatory and violent ways. It is not uncommon to read of magistrates and juries applauding the fool who comes forward and expresses his desire to take a criminal woman for the blessing of a priest and after the propagation of his and her kind in (save the words!) Holy Matrimony. Sentimentality allowing the criminal to dodge gaol by promising to try to propagate; and almost certainly succeeding in propagating.

It is earnestly to be hoped that the time is now near when the ratepayer and the taxpayer will have learnt that the lunatic, the feeble-minded person, and the most of the frequenters of our gaols are born and not made, and that the weighty charge on the community such persons entail is not a necessary charge, but an expense that can be got rid of by adequate measures. This heavy tax on industry can be steadily and surely reduced by efficient control of births.

